# Improving the Mechanical Properties of Hot Rolled Low-Carbon Copper-Containing Steel by Adjusting Quenching Roll Speed

**DOI:** 10.3390/ma17122953

**Published:** 2024-06-17

**Authors:** Henglin Wang, Ruiyang Chen, Xiaobing Luo, Zijian Wang, Hanlin Ding, Feng Chai

**Affiliations:** 1 School of Iron and Steel, Soochow University, Suzhou 215006, China; 20224049003@stu.suda.edu.cn (H.W.); 20214249001@stu.suda.edu.cn (R.C.); wangzijian@suda.edu.cn (Z.W.); 2 Department of Structural Steels, Central Iron and Steel Research Institute, Beijing 100081, China; luoxiaobing@cisri.com.cn (X.L.); chaifeng@cisri.com.cn (F.C.)

**Keywords:** low-carbon Cu-containing steel, quenching roller speed, Cu-rich precipitate, SAXS

## Abstract

This paper presents a comprehensive study of the impact of quenching roll speed on enhancing the low-temperature toughness of a low-carbon copper-containing steel. The microstructure characteristics, such as the prior austenite grains, and the distribution and volume fraction of precipitates, are observed using optical microscopy, scanning electron microscopy, transmission electron microscopy, and small-angle scattering X-ray. The results show that a decrease in the quenching roller speed (2 m/min) contributes to the achievement of more excellent low-temperature toughness (the average value is 232 J), although the prior austenite grains exhibit a relatively larger size in this case. The tempering treatment results in the precipitation of a large amount of 9R-type Cu-rich particles, regardless of the quenching roller speed. Reducing the quenching roller speed contributes to the increase in the volume fraction of Cu-rich particles, which is considered to be the main factor contributing to the achievement of excellent low-temperature toughness.

## 1. Introduction

In order to fulfill the stringent demands of the marine environment on structural materials, more attention has been paid in the development of marine engineering steel that has high strength, excellent low-temperature toughness [1,2,3,4,5,6,7,8], and good weldability [9]. During the preparation of marine engineering steel, the difficulty lies in the effective penetration of rolling forces, which is constrained by the thickness of the plate itself. Consequently, this often leads to significant disparities in the structural characteristics between the core and surface of the plate [1,2,3]. Zhang, Pallaspuro, Lin and Wang et al. have demonstrated that the precipitation of specific alloy elements such as Cu, Ni, Nb, Ti, Mo, and Cr in the form of specific precipitated phases can effectively retard grain growth and enhance the mechanical properties of the steel [4,5,6,7]. It is well known that the reduction of carbon content can significantly improve the weldability of steels, while grain refinement has been demonstrated as one of the most efficient approaches for simultaneously enhancing strength and toughness. 

As compared to traditional water quenching methods, the roller quenching technology offers improved efficiency and greater application prospects by adjusting the speed of the roller to achieve different holding times during the cooling process after hot rolling [10]. However, it is challenging for heavy plates produced by this method to achieve a uniform distribution of grain size along the thickness direction. This phenomenon exhibits a tendency to become more pronounced as the roller speed increases. The presence of an uneven distribution of grain size can result in variations in the mechanical properties between the surface and center of the rolled plate, ultimately leading to a decline in low-temperature toughness [10,11]. It has been reported that alloying elements such as Cr, Ni, and Mo can significantly improve the hardenability of steel [6,12,13,14]. Trace addition of these elements has the potential to enhance microstructural homogeneity across the thickness of the plate. Nevertheless, the escalation in the cost of raw materials, the increased carbon equivalent, and the potential segregation of alloying elements are emerging concerns that must not be overlooked [15,16,17]. It is evident that this is not conducive to enhancing the low-temperature toughness and weldability of marine engineering steels.

Recently, there has been a significant increase in research focusing on low-alloying Cu-containing marine engineering steels, which has garnered considerable attention in the field. Wang et al. [18] discovered that the inclusion of Cu in the steel composition led to a notable enhancement in low-temperature toughness. Specifically, the impact energy at −40 °C for the steel with a 1.5% Cu addition was approximately twice as high as that of the steel without any Cu addition. Moreover, the presence of Cu-rich precipitates contributes to the dispersion strengthening effect, compensating for the decline in mechanical properties resulting from the decrease in carbon content [19,20,21,22]. A design approach emphasizing a low carbon composition was employed by Ghosha and Jiang et al. The carbon content of the experimental steel was within the range of 0.03~0.05%, while Cu elements were incorporated in the range of 1.0~2.0% [19,20]. This approach ensures the weldability of the low-carbon steel while facilitating the precipitation of Cu-rich phases, thereby enabling the steel plate to achieve a yield strength exceeding 1000 MPa, accompanied by consistent impact toughness. However, it is noteworthy that this study differs from previous research, which typically utilized significant compression deformation in conjunction with corresponding heat treatment techniques to achieve finer grain sizes and consequently higher strength. In contrast, the present study exhibits slightly lower performance due to the thicker plate, which limits the compression ratio and, subsequently, the degree of tissue refinement. Additionally, Cu addition has been found to have an impact on the diffusion of alloying elements and variant selection. This effect leads to a reduction in element segregation at grain boundaries, thereby inhibiting the coarsening of grains in the heat-affected zone of welded joints [18,23,24]. 

In addition to the micro-alloying, several studies have been conducted to optimize the rolling process parameters in order to enhance microstructural homogeneity and mechanical properties [10,11,25,26,27]. For instance, Fu et al. [10] found that reducing the temperature of the quenching water and/or decreasing the roller speed can enhance the hardenability of the steels by reducing the temperature gradient along the thickness direction. The authors also proposed that the control of roller speed exerts a more significant influence on the cooling rate compared to the temperature of quenching water. Based on the simulated results obtained from DEFORM-3D 7.2 software, Bian et al. [11] observed that the cooling rate in the central region along the thickness direction is comparatively lower than that at the surface. Consequently, this disparity in cooling rates led to a significant difference in the average grain size at 1/4 and 1/2 thickness.

To enhance production efficiency, it is crucial to prioritize high-quality production. Nevertheless, despite the potential for production efficiency to be enhanced through the augmentation of quenching roll speed, the underlying cause for the significant disparity in the low-temperature impact toughness of the steel plate remains relatively unexplored. This paper delves into and meticulously examines the challenges encountered during the enhancement of quenching roller speed, thereby pinpointing the precise causes to establish a solid foundation for subsequent high-quality production endeavors.

## 2. Materials and Methods

Considering the potential issues associated with welding in practical applications, an ultra-low-carbon design for the studied steel was adopted in our work. The corresponding chemical compositions are illustrated in Table 1. A certain amount of Cu was added with the aim of forming nano precipitates during heat treatment, thereby compensating for the strength deficiency caused by the ultra-low-carbon design. The addition of Ni aims to improve the toughness of the steel, especially at low temperature.

The ingots were reheated to 900 °C and then held for 1 h before hot rolling. The steel was hot-rolled to 26 mm-thick sheets with different roller speeds of 2 m/min and 6 m/min. Previous experimental investigations have demonstrated that the temperature of 900 °C falls below the non-recrystallization threshold of the experimental steel with the given alloy composition. In the context of fabricating thick plate steel, rolling at a temperature below the non-recrystallization point is more beneficial for accumulating strain, as it effectively prevents the dissipation of accumulated strain due to dynamic recrystallization of austenite during the rolling process. This approach facilitates the refinement of the microstructure and enhances the overall strength of the steel. The rolled sheets were cooled to room temperature (RT) by water spraying immediately after hot rolling. The amount of water spraying was controlled at a constant value of 30 L/(m^2^·s). The rolled plates were then subjected to tempering at 660 °C for 2 h, followed by air cooling to RT. Hereafter, the samples cut from different roller speeds (2 m/min or 6 m/min) and heat treatment states (quenching or tempering) will be referred as 2Q, 6Q, 2T, and 6T, respectively.

Samples with a size of 10 mm × 10 mm × 26 mm were utilized for microstructure observation, aiming to analyze the variation in microstructural differentiation from the surface to the center of the rolled plates. Vickers microhardness measurements were conducted at an applied loading of 500 g and subsequently held for 15 s at RT. Charpy impact specimens were machined into standard V-notch samples with dimensions of 10 mm × 10 mm × 55 mm (V-notch depth: 2 mm) and then tested at −84 °C.

The microstructure of the samples was analyzed using an optical microscope (OM, Axio Vert. A1, ZEISS Ltd., Oberkochen, Germany), a scanning electron microscope (SEM, SU-5000, Hitachi Ltd., Tokyo, Japan), and a transmission electron microscope (TEM, F200X, FEI Talos Ltd., Waltham, MA, USA) equipped with an energy dispersive X-ray detector (EDX) at 200 kV. The high-angle annular dark field (HAADF) image of each alloy element was further observed by STEM-HAADF scanning transmission. The nanosized precipitates were also evaluated by using a small-angle X-ray scattering (SAXS, SAXSess MC2, Anton Paar Ltd., Graz, Austria) at a voltage of 40 kV.

The specimens for OM and SEM analyses were mechanically polished using SiC paper of up to #2000 grit. The specimens were soaked into a saturated picric acid solution for 120 s at 65 °C to achieve the prior austenite grain boundaries (PAGBs). The specimens for SEM were etched with a 4 vol.% nitrate alcohol solution. The thin foils used for TEM observations were processed by wire-cut electrical discharge machining (WEDM) to attain a thickness of 1 mm, mechanically thinned to ~70 μm using sandpaper of 80#, 600#, 2000#, and 4000#, and twin-jet electropolished in an electrolyte of 10 vol.% perchloric acid and 90 vol.% ethanol at a temperature below −25 °C and an electrolytic voltage of 20 V. The light sensitivity value was adjusted to 10 to ensure optimal conditions. The TEM sample preparation was deemed complete when a minute hole of approximately 0.2 mm in diameter appeared centrally in the thin foils, in preparation for TEM observations. The SAXS testing conditions were standardized at a voltage of 40 kV and a current of 50 mA. As for sample preparation, the approach mirrored that utilized for TEM samples, involving mechanical thinning to approximately ~100 µm, while omitting the electrolytic polishing step to facilitate direct testing.

## 3. Results and Discussion

### 3.1. Mechanical Properties

**Figure 1:**
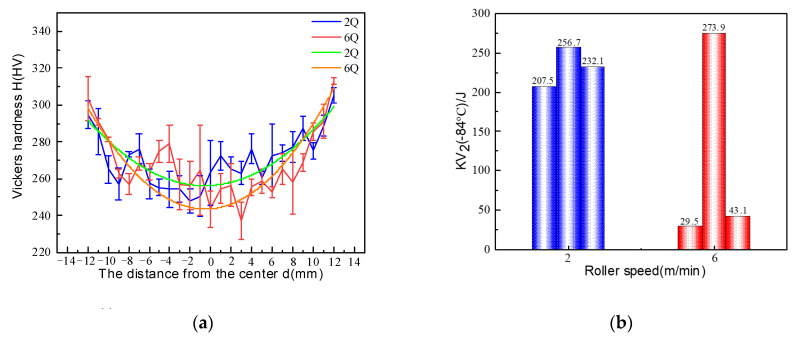
The distribution of the Vickers hardness along the direction of the rolled plate thickness (**a**) and the impact energy of the rolled plates after tempering (**b**).

Figure 1 shows the mechanical properties of the studied steels. The variation of the Vickers microhardness as a function of the distance in the direction of the rolled plate thickness is illustrated in Figure 1a. It can be found that the Vickers hardness exhibits a decreasing trend with distance from the surface until it reaches a minimum value. This trend is observed in both plates that were rolled at different roller speeds. All of these minimum values appear in the central region of the thickness direction, indicating significant microstructure differentiation in the rolled plates. Additionally, the Vickers hardness of sample 6Q generally exhibits a relatively lower value when compared to that of sample 2Q, especially in the central region of rolled plates. Figure 1b shows the impact energy at −84 °C of the studied steel after tempering. The impact energy values for 2T samples are 207.5 J, 256.7 J, and 232.1 J, whereas for 6T samples, the impact energy values are 29.5 J, 273.9 J, and 43.1 J, respectively. Obviously, the impact toughness of sample 2T demonstrates a relatively consistent stability when compared to sample 6T. As well known, the homogeneity of microstructure plays a crucial role in determining the impact toughness [22]. The fact that the impact toughness of sample 2T is superior to that of sample 6T can be attributed to the influence of the roller speed on the microstructure characteristics.

**Figure 2:**
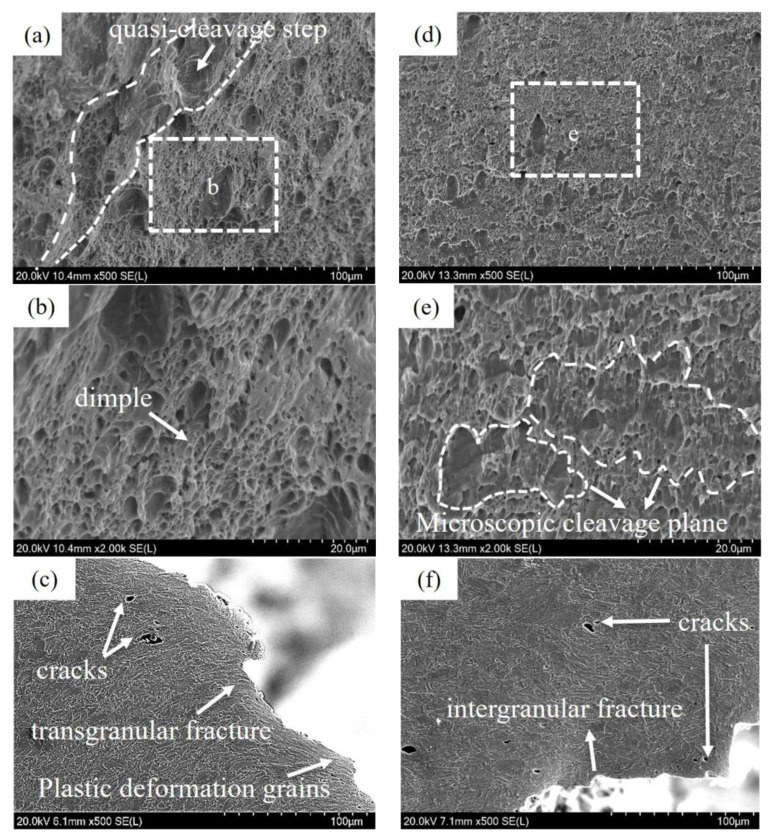
Impact fracture morphology of 2T and 6T samples: (**a**) crack initiation origin of 2T; (**b**) Figure 2b shows a magnified view of the dotted box part in Figure 2a, which shows the dimple feature; (**c**) the fracture side surface of crack initiation origin of 2T; (**d**) crack initiation origin of 6T; (**e**) Figure 2e shows a magnified view of the dotted box part in Figure 2d, magnification of crack initiation origin of 6T; (**f**) the fracture side surface of crack initiation origin of 6T. All photographs depicted in Figure 2 were captured using scanning electron microscopy (SEM) in the Secondary Electron mode, employing the HITACHI SU-5000 Scanning Electron Microscope.

Given the significant instability and variance in the impact energy that 6T samples exhibited, it has been deemed necessary to select a specific 6T sample with an impact energy of 29.5 J for comparative analysis with the 2T, which possesses an impact energy of 207.5 J. As depicted in Figure 2a, the quasi-cleavage step and tearing ridge of 2T were exhibited, extended radially along the direction of impact. Adjacent to these cleavage steps, numerous dimples were observed (Figure 2b), which arose from the microscopic plastic deformation of grains under heavy loads. The presence of a large number of dimples typically indicates superior impact toughness within the material. The crack initiation origin of 2T was excised perpendicularly, allowing for observation of the fracture side surface (Figure 2c). The characteristics of transgranular fracture were revealed, accompanied by a significant number of plastic deformation grains close to the fracture. A substantial portion of the impact energy was effectively absorbed by plastic deformation grains, and thereby the impact toughness of the material was enhanced. This observation aligns with the high and consistent value performance demonstrated by the 2T sample in Figure 1b. In contrast, the crack initiation origin morphology of 6T, as shown in Figure 2d,e, differed significantly from that of 2T. Notably absent were the dimples characteristic of micro-zone cleavage surfaces in brittle fracture. Intergranular fracture characteristics were exhibited in the crack initiation origin of 6T, and the grains adjacent to the fracture did not undergo appreciable plastic deformation. Consequently, 6T exhibited immediate failure, resulting in low and inconsistent impact energy. Factors influencing the impact energy of these samples may be linked to the uniformity of their microstructure and the distribution of particles.

### 3.2. Microstructure Characterization

#### 3.2.1. Prior Austenite Grain Boundaries

**Figure 3:**
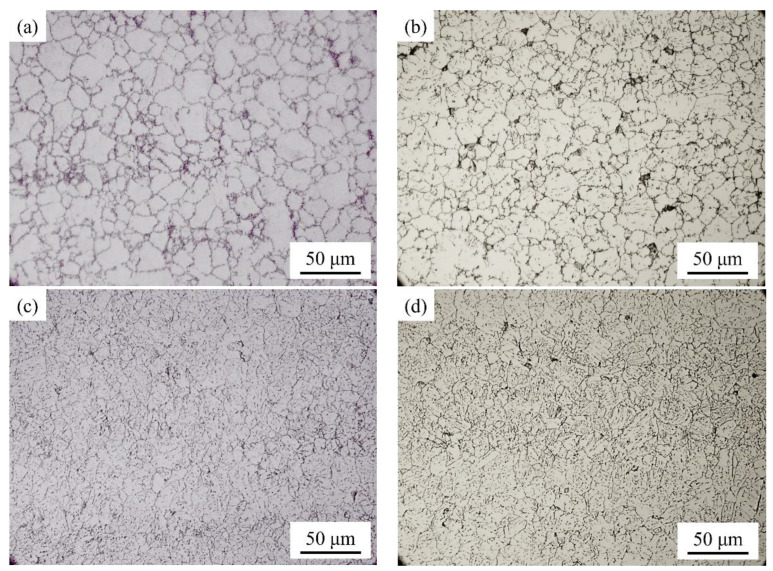
Optical micrographs of prior austenite grain boundaries for specimens 2Q and 6Q. (**a**) 2Q, near the surface of rolled plates; (**b**) 6Q, near the surface; (**c**) 2Q, the central region of the rolled plates; (**d**) 6Q, the central region. The figures (**a**–**d**) sampling position is designated for obtaining samples along the thickness direction of the steel.

To analyze the effect of roller speed on the microstructures of rolled plates, the prior austenite grains (PAGs) of the plates rolled at different roller speeds have been investigated. Optical micrographs of the PAGBs for specimens 2Q and 6Q are shown in Figure 3. The statistical results show that the average grain size is 10.9 ± 5.1 μm (Figure 3a) and 11.1 ± 5.6 μm (Figure 3c) for the PAGs near the surface and in the center of specimen 2Q, respectively. For specimen 6Q, the corresponding values are 10.3 ± 5.9 μm and 10.7 ± 5.4 μm, just as shown in Figure 3b,d. The size of PAGs in specimen 2Q is slightly larger than that in 6Q, which can potentially be attributed to the prolonged exposure to high temperature and subsequent grain growth in the 2Q plate due to its lower roller speed. Due to the same tempering condition, it can be speculated that the relative size of PAGs would not change significantly for the samples after tempering treatment. However, the slight difference in PAG size might not be the primary reason for the difference in impact toughness. Therefore, it is necessary to further analyze the microstructures and to clarify the reasons for the difference in mechanical properties.

#### 3.2.2. Microstructure Examinations via SEM and TEM

**Figure 4:**
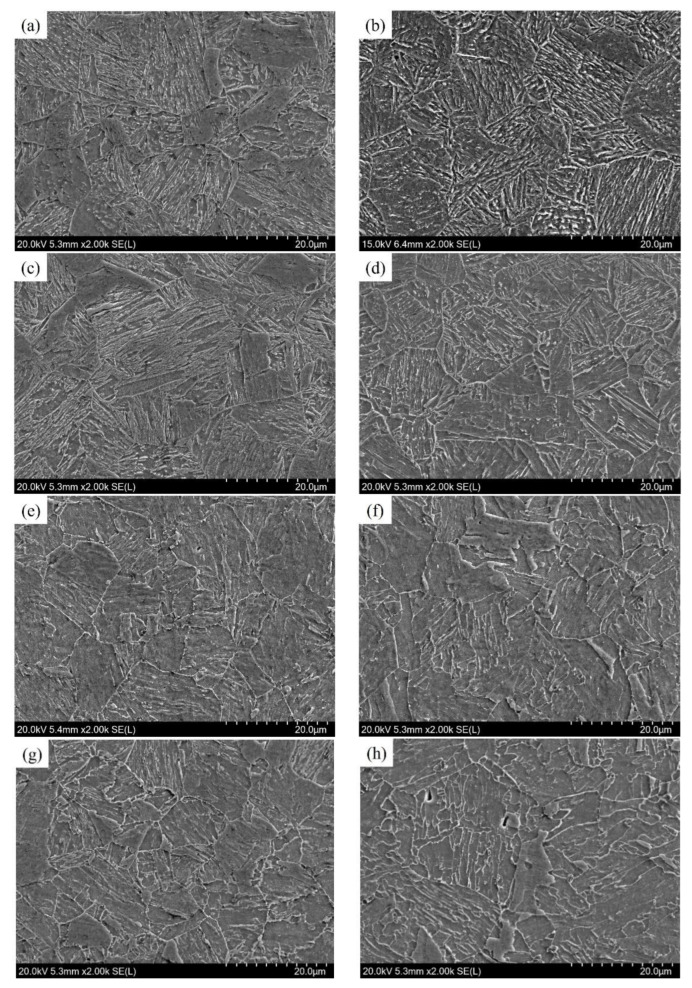
SEM micrographs showing the microstructures of quenched and tempered steel plates rolled at different roller speed: (**a**) 2Q, near the surface; (**b**) 2Q, the central region; (**c**) 6Q, surface; (**d**) 6Q, center; (**e**) 2T, surface; (**f**) 2T, center; (**g**) 6T, surface; (**h**) 6T, center. All photographs depicted in this figure were captured using scanning electron microscopy (SEM) in the Secondary Electron mode, employing the HITACHI SU-5000 Scanning Electron Microscope.

Figure 4 shows the SEM micrographs of the quenched and tempered steel plates rolled at different roller speeds. For the quenched samples, the typical martensite characteristics can be found, especially for the microstructures near the surface, just as shown in Figure 3a–d. Due to the influence of the plate thickness, the surface of the rolled plate usually has a higher cooling rate than the central region, which results in a significant temperature gradient along the plate thickness direction [10,11]. Once the actual temperature in the central region of the plate is maintained above the martensitic transition temperature, bainite transformation will inevitably occur [10]. This is also the primary reason that causes the microstructure variation from the surface to the center of the rolled plates. Furthermore, as a result of the different roller speeds, plate 2Q obtained a longer residence time in water than plate 6Q during the spray quenching process, which promoted more martensite transformation, especially along the direction of the plate thickness. As a result, for the specimen 2Q, a mixture of martensite and bainite can be found in the central region (Figure 4b), while it is a typical bainite for specimen 6Q (Figure 4d).

A further investigation into the microstructure evolution of as-quenched samples during tempering is illustrated in Figure 4e–h. It is evident that tempering treatment leads to the decomposition of the microstructure, causing the martensite lath characteristic to weaken or disappear when compared to the as-quenched plates (Figure 4a–d). Especially for the microstructures near the surface of tempered plates (Figure 4e,g), the typical martensite laths, regardless of whether the specimens are 2T or 6T, tend to be difficult to distinguish. Perhaps due to the low carbon content, 0.033%, in the studied steel, there are almost no obvious granular carbide precipitates observed in these SEM micrographs.

**Figure 5:**
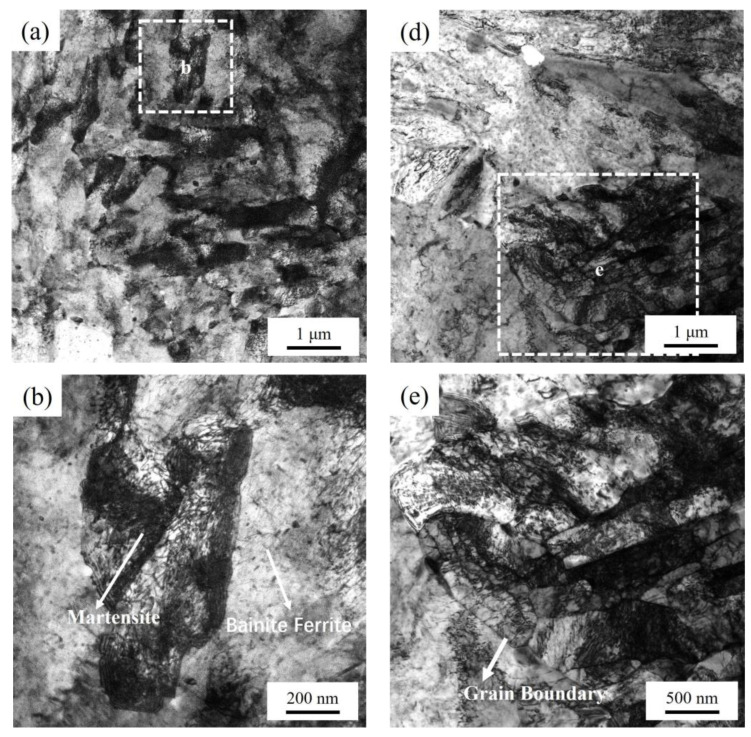

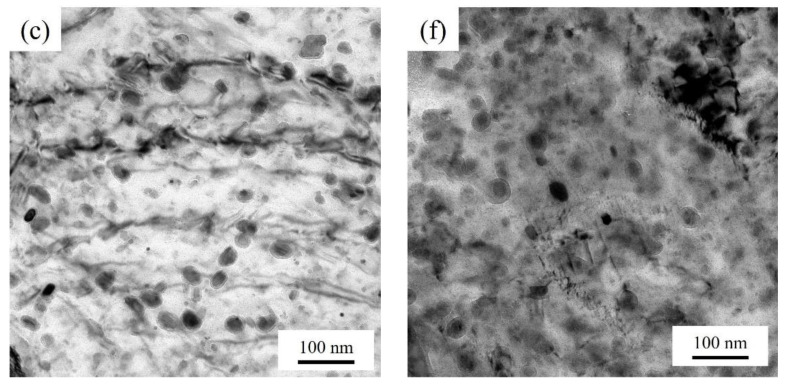
BF-TEM micrographs of the as-tempered steels. Dislocation image figures (**a**,**b**) presents a detailed local enlargement of the region enclosed by the white dashed box depicted in (**a**), and the precipitates (**c**) in the tempered sample 2T; dislocation image figures (**d**,**e**) offers a similarly magnified view of the area outlined by the white dashed box in (**d**) and the precipitates (**f**) in the tempered sample 6T.

TEM images showing both grain boundaries and grain interior regions of as-tempered samples are presented in Figure 5. It can be clearly observed that the martensite decomposition took place during the tempering. Some of the tempered martensite still retained a lath-like morphology and contained a high density of dislocation, as shown in Figure 5b,e. The tempered martensite observed in sample 2T (Figure 5a) appears to exhibit a smaller size and a more uniform distribution compared to that in sample 6T (Figure 5d). In addition, those decomposed regions primarily consist of bainite ferrite and ultrafine dispersed particles, such as carbides and other precipitates. Such particles are not easily observed in the tempered martensite due to the existence of a large number of dislocations. These particles with nanoscale size usually exhibit distinct spherical characteristics and uniformly distribute on the bainite ferrite (Figure 5c,f). It is noted that some dislocations entangled with the precipitates can be found in the ferrite matrix of sample 2T (Figure 5c), while they are barely found in sample 6T (Figure 5f). 

#### 3.2.3. Precipitates and Their Distribution in As-Tempered Specimens

**Figure 6:**
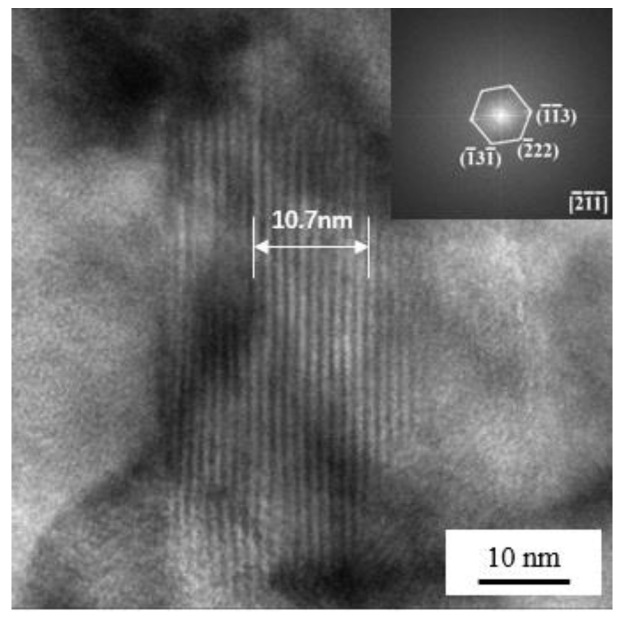
The HRTEM images of typical Cu-rich precipitate in sample 2T; the upper right plot is the Fast Fourier Transform (FFT) plot of the whole image.

Figure 6 shows the high-resolution transmission electron microscopy (HRTEM) images depicting the typical particles observed in sample 2T. The results obtained from the upper right fast fourier transform (FFT) plot of the whole image inset in Figure 6 confirm that these particles with twin morphologies can be identified as Cu-rich precipitates. These Cu-rich particles with sizes of 20 nm have a non-coherent state with the matrix. The thickness of 10 lamellar structure is 10.7 nm, with each layer having a thickness of 1.07 nm. Each layer is composed of four atoms, with a lattice parameter of d = 0.357 nm, indicating a typical 9R Cu structure. Sun et al. [28,29] reported that type-9R Cu has the ability to undergo a direct transition to a twin fcc Cu structure, without any intermediate transitions. It has also been proposed that the defects, such as dislocations, can serve as nucleation sites for the precipitation of Cu particles or provide effective diffusion channels for the metal atoms during the tempering process. This phenomenon is believed to reduce the nucleation barrier and subsequently enhance the nucleation of Cu particles [28,30]. Obviously, sample 2T is expected to exhibit a higher dislocation density compared to sample 6T. This can be attributed to the lower quenching speed of sample 2T, which results in a stronger quenching ability. As shown in Figure 4c,f, the dislocation density in sample 2T is comparatively higher than that in sample 6T. Consequently, the diffusion of Cu atoms is more sufficient and easier in sample 2T, resulting in the formation of finer Cu-rich particles. This, in turn, contributes to the enhancement of both strength and toughness.

**Figure 7:**
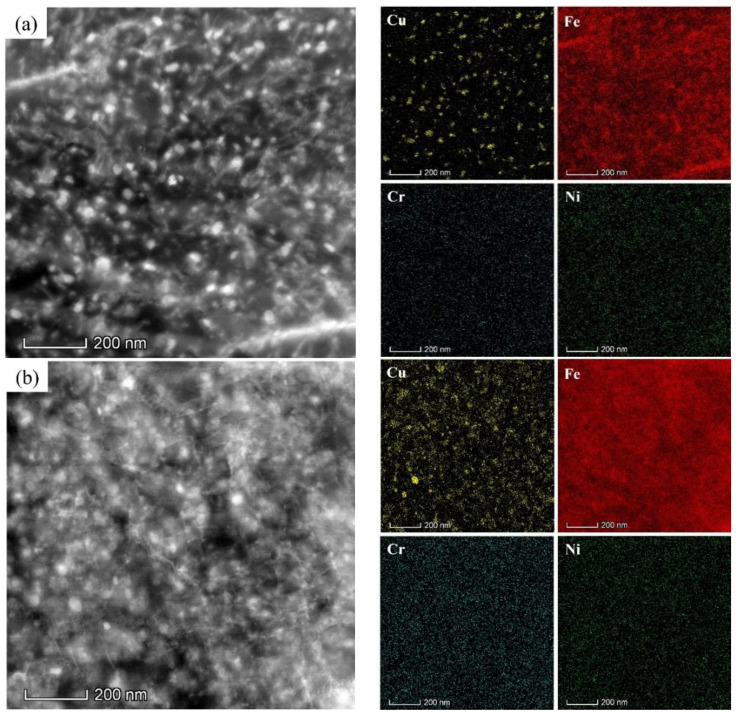
STEM-HAADF observation and EDS spectrum. (**a**) 2T of precipitates after tempering of test steel; (**b**) 6T of precipitates after tempering of test steel.

To further explore the difference in the precipitates between sample 2T and 6T, the morphology observations and the corresponding EDS analysis are characterized by STEM. The distribution of nanoscale precipitates and the atom maps of Cu, Fe, Cr, and Ni elements are illustrated in Figure 7. The EDS analysis reveals that large amounts of spheroidal precipitates with a diameter less than 20 nm in both samples are enriched in Cu element. Therefore, it can be concluded that the Cu-rich-type precipitates are the primary strengthening phases in the studied steel after tempering. Note that the difference between the two types of samples in the distribution of Cu element in the matrix cannot be ignored. That is, the enrichment of Cu element in sample 2T is more pronounced than that in sample 6T, so that the outline of nanoscale Cu precipitates can be observed more clearly in sample 2T (Figure 7a), while the distribution of Cu atom in sample 6T appears relatively dispersed (Figure 7b). As mentioned above, due to the low quenching roll speed, the typical microstructure in sample 2T is martensite, in which the solute atoms, including Cu, tend to be supersaturated and then provide more driving force for the nucleation of precipitates. In addition, the dislocation density resulting from the shear deformation during martensite transformation will accelerate the precipitation of Cu-rich particles. While the original intention of this study is to enhance production efficiency during the hot rolling of studied steel, our premise is that excellent comprehensive mechanical properties can still be obtained. However, the results above show that the higher roll speed are crucial for the improvement of mechanical properties of studied steel due to the insufficient precipitation of Cu-rich particles.

Small-angle X-ray scattering (SAXS) possesses the capability to swiftly gather information on precipitations of the test sample across a vast area. In comparison to transmission electron microscopy (TEM), SAXS exhibits noteworthy benefits in evaluating the characteristics of precipitations within a specific region, encompassing factors such as morphology, volumetric fraction, higher efficiency, and lower testing cost. SAXS measurement is a well-established technique for the characterization of nanoparticles with respect to the volume fraction of particles. SAXS data for precipitates in samples 2T and 6T are displayed by the electron scattering intensity *I* and scattering vector *q* in Figure 7. The curves contain information about the volume fraction of precipitates, which can be described as [31]:

(1) $$Q_{0}=\int_{0}^{\infty }Iq^{2}dq=2\pi^{2}f_{v}\left(1-f_{v}\right){\left(\Delta \rho \right)}^{2}$$

where *Q*_0_ is the integrated intensity in the SAXS curves, which can be calculated based on the volume fraction between the precipitate (*f_v_*) and the matrix (1 − *f_v_*), while it is independent of the precipitate’s size. Δ*ρ* is the difference in scattering length density between the precipitate and the matrix. It can be achieved by the following equation:

(2) $$\Delta \rho =\rho_{p}-\rho_{m}=\frac{z_{p}}{\Omega_{p}}-\frac{z_{m}}{\Omega_{m}}$$

where *Z_p_* and *Ω_p_* are the atomic number and atomic molar volume of the precipitate, respectively, while *Z_m_* and *Ω_m_* are the atomic number and atomic molar volume of the matrix, respectively.

**Figure 8:**
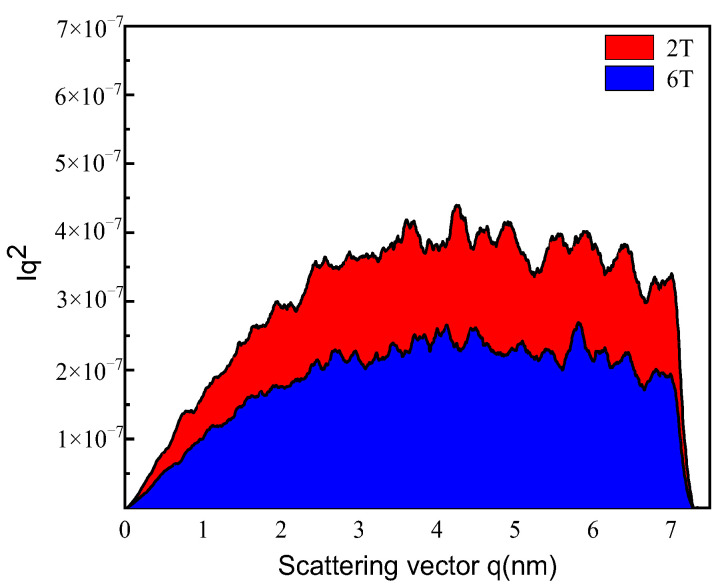
Plots of electron scattering intensity, *I*, versus scattering vector, *q*, from the SAXS results of samples 2T and 6T.

The integrated intensity *Q*_0_ can be derived from the curves depicted in Figure 8, which can be quantified by calculating the area under the *Iq*^2^ curves. It is clear from Figure 8 that the total amount of precipitates characterized by integrated intensity in sample 2T is significantly higher than that in sample 6T, showing a remarkable agreement with the TEM observations in Figure 7. The calculation methodology for determining the integrated intensity *Q*_0_ and the volume fraction of precipitates is detailed in Refs. [31,32,33]. The calculated values of *Q*_0_ for samples 2T and 6T are 2.20 and 1.32, respectively. According to the further analysis presented in Equation (1), the estimation of the volume fraction derived from SAXS data is determined to be 0.63% and 0.38% for samples 2T and 6T, respectively. Based on TEM observations presented in Figure 6 and Figure 7, all the precipitates can be assumed to be Cu-rich particles. Note that the anticipated total volume fraction, based on the alloy composition of the studied steel, is expected to be ~1.1%. That is, the volume fractions of precipitates measured by SAXS are found to be lower than what would be expected based on the alloy composition. Due to the inherent constraint of the SAXS scanning angle range, certain particles with larger dimensions may not be effectively captured, resulting in a measured volume fraction of precipitates that is lower than the theoretically predicted maximum volume fraction. An additional plausible explanation for this result is that there are still some Cu atoms dissolved in the matrix, which will exert the effect of solid solution strengthening for the studied steel. Therefore, in order to further enhance the mechanical properties of studied steel, it is possible to optimize process parameters such as roller speed, cooling rate after hot rolling, and holding time in tempering. This optimization aims to accelerate the precipitation of Cu-rich particles. Of course, the strict consideration of suppressing the coarsening of Cu-rich particles is essential in the design of process parameters.

## 4. Conclusions

We have systematically studied the microstructure and precipitates in a low-carbon Cu-containing steel obtained at different quenching roller speeds. The relationship between the microstructure and toughness was discussed. The following conclusions can be drawn:

(1) Reducing the roller speed leads to an increase in the prior austenite grain size, while enhancing the hardenability depth along the thickness direction of the rolled sheet forms a larger volume fraction of martensite. The rolled sheet prepared at lower quenching roller speed has a higher hardness value and excellent toughness at low temperature.
(2) The tempered martensite is the dominant characteristic of the microstructure of as-tempered steels. Significant quantities of Cu-rich particles are observed to precipitate during the tempering treatment, regardless of the quenching roller speed. TEM analysis confirms that the particles rich in Cu have a typical 9R structure.
(3) In comparison to the steel produced under a quenching roller speed of 6 m/min, the steel prepared at 2 m/min exhibits a higher density of dislocations and Cu-rich particles. The estimated volume fraction of Cu-rich particles based on SAXS data is approximately 0.63% for sample 2T and 0.38% for sample 6T. The primary factor contributing to the achievement of excellent low-temperature toughness is the precipitation of a higher volume fraction of Cu-rich particles.

## Figures and Tables

**Table 1:** The chemical compositions of the investigated steel (wt.%).

C	Si	Mn	Cr	Ni	Cu	Mo	Ti	Nb	Fe
0.033	0.22	0.57	0.66	1.3~1.6	1.1~1.4	0.15~0.35	0.016	0.030	Bal.

## Data Availability

The original contributions presented in the study are included in the article, further inquiries can be directed to the corresponding author.
